# A Single-Center, Randomized, Double-Blind, and Placebo-Controlled Comparative Human Study to Verify the Functionality and Safety of the *Lespedeza cuneata* G. Don Extract for the Improvement of Aging Male Syndrome

**DOI:** 10.5152/tud.2023.23065

**Published:** 2023-09-01

**Authors:** Hyungjee Kim, Yu-Mi Seo, Seong Lee, Dahye Kang, Changhee Kim

**Affiliations:** 1 Department of Urology, Dankook University Medical College, Cheonan, Republic of Korea; 2 Research Institute of Clinical Medicine, Dankook University Hospital, Cheonan, Republic of Korea; 3 R&D Center, Naturalway Co., Ltd., Pocheon, Republic of Korea

**Keywords:** Aging, androgen, andropause, male, *Lespedeza cuneata*

## Abstract

**Objective:**

Aging male syndrome is a clinical biochemical syndrome characterized by typical aging symptoms and serum testosterone deficiency. Although it is accompanied by various health problems, directly affects life satisfaction, and requires proper management, no clear prevention or treatment other than hormone replacement therapy is currently available for this syndrome. Here, we aimed to determine the efficacy and safety of the *Lespedeza cuneata* extract in the management of the aging male syndrome.

**Methods:**

Males aged 43-70 years who provided consent for participation and had a total Aging Males’ Symptom questionnaire score ≥ 37 and testosterone level ≤ 500 ng/dL were enrolled in this study. This study was conducted in a randomized, double-blind manner. Participants were randomly assigned to either the experimental or control groups and orally administered the assigned product twice a day. Efficacy was evaluated by measuring changes in Aging Males’ Symptom score, Androgen Deficiency in the Aging Male questionnaire score, International Index of Erectile Function score, International Prostatic Symptom Score, blood test results, and body mass index at 8 weeks.

**Results:**

After 8 weeks, the experimental group had significantly improved symptom scores compared to the control group on the Aging Males’ Symptom and Androgen Deficiency in the Aging Male questionnaires. However, no significant differences in the International Index of Erectile Function score, International Prostatic Symptom Score score, blood test results, and body mass index were observed between the experimental and control groups.

**Conclusion:**

*Lespedeza cuneata* extract safely alleviates andropause symptoms without any significant side effects, suggesting its potential for the treatment of the aging male syndrome.

**Main Points**

- *Lespedeza cuneata* extract (LCE) had significantly improved symptom scores on the Aging Males’ Symptom and Androgen Deficiency in the Aging Male questionnaires.
- Significant changes had not been observed in the safety assessment between the control group and the experimental group.
- This study concludes that LCE has the potential for the treatment of the aging male syndrome without any significant side effects.

## Introduction

Andropause syndrome is a clinical and biochemical syndrome characterized by typical symptoms such as decreased cognitive ability, spatial orientation, fatigue, depression, and mood changes. It occurs due to old age and serum testosterone deficiency.^1,2^ The prevalence of symptomatic androgen deficiency is 5.6% in men aged 30-79 years and increases substantially with age.^3^

Andropause, a condition characterized by decreased serum testosterone levels, can be partially managed via lifestyle changes such as maintaining a healthy diet, getting sufficient rest, and doing regular exercise and strength training. Testosterone replacement therapy (TRT) is an important treatment option for this syndrome that can be administered via injection, oral preparations, and transdermal gels. Injections should be administered slowly to prevent adverse side effects such as acne, facial flushing, depression, anxiety, and abnormal liver function tests. Oral preparations need to be administered multiple times daily, which may cause jaundice, gynecomastia, and edema. Transdermal administration of testosterone is another treatment option that requires regular monitoring of serum testosterone levels to ensure proper dosage and frequency of the gel. Despite its potential side effects, TRT is the primary treatment for andropause, as no other prevention or treatment options are currently available for this condition.^4^

Natural products have gained increasing interest as alternative therapeutic agents for andropause owing to the potential adverse effects of TRT. The MR-10 complex extract (dandelion and fermented leaves of rooibos) and a mixed extract of fenugreek seeds and *Lespedeza cuneata* are used as functional raw materials for andropause treatment as they alleviate andropause symptoms and improve overall male health without any side effects. These natural products improve testosterone levels, enhance sexual function, reduce fatigue and depression, and promote the overall well-being of the affected males.^5-7^ Therefore, further exploration of natural products can aid in the development of safe and effective treatment strategies for andropause.

*L. cuneata* G. Don, a vascular plant belonging to the Fabaceae family, is a perennial herb with shrub properties that typically grows in lowland grasslands and fields.^8^ The nourishing effects of* L. cuneata *on the liver and lungs and its alleviation of symptoms related to male menopause have been detailed in Oriental medical literature.^9,10^ *L. cuneata *contains many physiologically active substances, such as pinitol, flavonoids, phenol compounds, and β-sitosterol. Among the flavonoids, quercetin, kaempferol, vitexin, and desmodin have been reported in previous studies.^10,11^ A preclinical experiment reported that the oral administration of the *L. cuneata* extract (LCE) not only alleviated the symptoms of aging but also increased the serum levels of testosterone in aged rats (34-weeks-old).^12^ Here, we designed a clinical study to confirm the safety and efficacy of LCE in alleviating the andropause syndrome.

## Material and Methods

### Study Design

This study was approved by the Institutional Review Board (IRB) of Dankook University Hospital (IRB Number: DKUH 2020-08-018) and conducted in accordance with the International Council for Harmonisation of Technical Requirements for Pharmaceuticals for Human Use guidelines and ethical principles of the Declaration of Helsinki. Informed consent was confirmed by the IRB.

This study was a double-blind, randomized, placebo-controlled, and parallel-group study. Patients were prospectively and consecutively selected between August 2020 and August 2021. Only the eligible patients after the screening test were randomly assigned to either the experimental or control (placebo) group at the first visit (visit 1) and instructed to take their respective products orally for 8 weeks. After the ingestion of the experimental and control products, the patients were evaluated for any changes at the end of the eighth week.

### Subjects

Male subjects were eligible if they were aged between 43 and 70 years and had a total score ≥ 37 on the Aging Males’ Symptoms (AMS) questionnaire during screening and a total testosterone level ≤ 500 ng/dL in the blood test. Before randomization, the qualified subjects who voluntarily agreed to participate in the study and provided written informed consent to comply with the clinical trial guidelines were included in the study.

Exclusion criteria for this study included the following: serum alanine aminotransferase or aspartate aminotransferase levels > 100 IU/L, gamma-glutamyltransferase levels > 100 IU/L, serum creatinine levels > 1.4 mg/dL, uncontrolled psychiatric disorders, body mass index (BMI) ≥ 35 kg/m^2^, prostate-specific antigen (PSA) levels ≥ 4.0 ng/mL, and alcoholism. Additionally, patients who had taken phosphodiesterase-5 inhibitors, donated whole blood, or received blood transfusions, androgen or anti-androgen medications, or systemic corticosteroids within the past 2 months were excluded from the study. Patients with a blood pressure ≥ 160/100 mmHg or fasting blood sugar level ≥180 mg/dL were also excluded from the study. Those who had started new medications for diabetes within the past 3 months had a total score ≥ 20 on the International Prostate Symptom Score (IPSS) questionnaire, or had participated in other clinical trials within the past 2 months were also excluded from the study.

### Experimental Products

Experimental and placebo products were orally administered twice daily to the subjects. A single tablet of the experimental product was 800 mg containing 625 mg LCE powder (LCEP), and the daily requirement was 2 tablets containing 1250 mg of LCEP. In the placebo group, dextrin was administered instead of the LCEP. Each product was consumed for 8 weeks, starting from visit 1 (baseline week 0).

### Outcome Measurements

Before starting the medication, several baseline measurements, including age, blood pressure, comorbidities, IPSS, International Index of Erectile Function (IIEF) score, AMS score, Androgen Deficiency in the Aging Male (ADAM) questionnaire score, homeostatic model assessment of insulin resistance (HOMA-IR) values, serum testosterone, sex hormone-binding globulin (SHBG), serum fasting glucose, total cholesterol, low-density lipoprotein (LDL) cholesterol, high-density lipoprotein (HDL) cholesterol, triglycerides, and insulin levels, were taken. The same measurements were repeated after 4 and 8 weeks of treatment to evaluate the efficacy of the experimental product.

Primary efficacy assessment measured any changes in the AMS, ADAM, IIEF, and IPSS questionnaire scores from baseline. Secondary efficacy assessment measured the changes in the serum testosterone, SHBG, serum fasting glucose, serum cholesterol (total, LDL, and HDL), triglyceride, and insulin levels and HOMA-IR values from the baseline. For safety evaluation, changes in blood urea nitrogen/creatinine (BUN/Cr) and PSA levels were measured, and urine analysis, complete blood count (CBC), and liver function tests were performed.

### Statistical Analysis

All values are represented as the mean ± standard deviation. Statistical analyses were conducted using the R statistical program version 3.4.4 (Foundation for Statistical Computing, Vienna, Austria). Differences among groups were evaluated using* t*-tests, chi-square tests, and repeated-measures analysis of variance. Statistical significance was set at *P *< .05.

## Results

### Characteristics of Subjects

A total of 103 men aged 43-70 years who scored ≥37 on the AMS questionnaire and had a testosterone level ≤ 500 ng/dL at a single institution participated in this study after providing written consent. Baseline characteristics of the participants did not differ significantly between the experimental and control groups (Table 1).

### Evaluation of the Primary Efficacy

**Figure 1.**
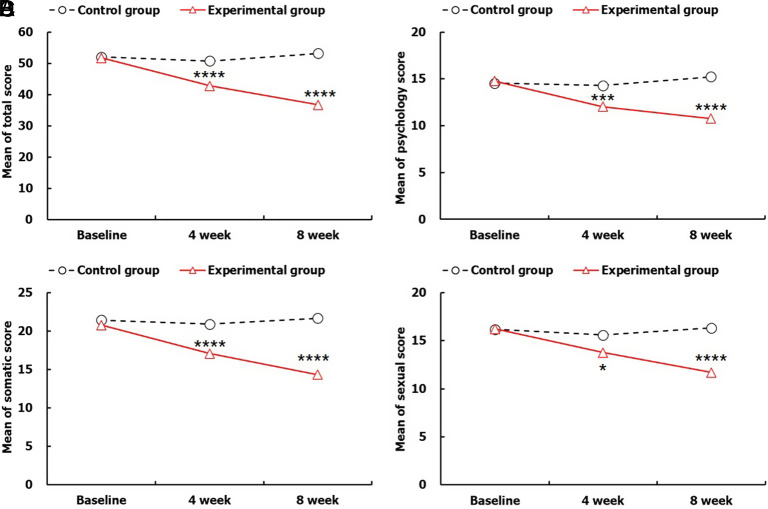
Changes of Aging Male's Symptom (AMS) survey: (A) the mean of total score, (B) the mean of psychology score, (C) the mean of somatic score, and (D) the mean of sexual score. Statistical differences between experimental group and control group were identified by repeated measure analysis of variance (****P < .0001, ***P < .001, **P < .01, and *P < .05 vs. Control group).

No significant differences in the baseline total AMS scores were observed between the experimental and control groups. The control group did not show significant differences in the total AMS scores 4 and 8 weeks after placebo administration. In contrast, the experimental group showed significant differences in the AMS scores 4 and 8 weeks after administration of the experimental product. Moreover, significant differences were observed between the 2 groups 4 and 8 weeks after administration of each product (Figure 1A; Table 2).

No differences in the psychological, somatic, and sexual scores were observed between the experimental and control groups at baseline. However, significant differences were observed between the 2 groups 4 and 8 weeks after treatment (Figure 1B–D; Table 2).

**Figure 2.**
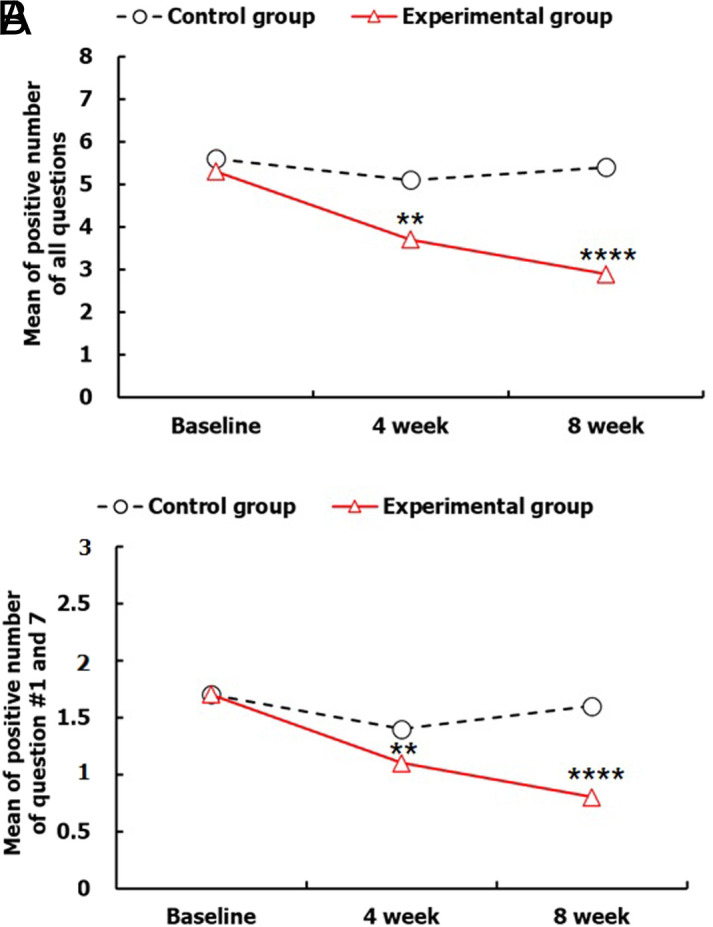
Changes of Androgen Deficiency in the Aging Male (ADAM) survey: (A) the mean of positive number of all questions and (B) the mean of positive number of questions 1 and 7. Statistical differences between experimental group and control group were identified by repeated measure analysis of variance (****P < .0001, ***P < .001, **P < .01, *P < .05 vs. Control group).

The ADAM questionnaire score in the experimental group was 7.0 ± 2.1 at baseline, which decreased significantly to 4.8 ± 3.3 at 4 weeks and 3.8 ± 3.3 at 8 weeks after administration. No significant difference was observed in the control group. The differences between the 2 groups were significant 4 and 8 weeks after the administration of the experimental product (Figure 2A; Table 3).

In the sexual questions (items 1 and 7) of the ADAM questionnaire, no statistically significant differences were observed in the average number of “yes” responses between the experimental and control groups at baseline and 4 weeks after treatment. However, a significant difference between the 2 groups was observed 8 weeks after treatment (Figure 2B; Table 3).

IIEF scores significantly increased at 4 and 8 weeks compared to the baseline in the experimental group, whereas no significant change was observed in the control group at 8 weeks. Moreover, no significant differences were observed in the IIEF scores between the two groups at 8 weeks (Table 4).

Both the experimental and control groups showed a significant decrease in IPSS and quality of life scores at 4 and 8 weeks. However, no significant difference was observed between the 2 groups at 8 weeks (Table 5).

### Evaluation of the Secondary Efficacy

No statistically significant differences in the laboratory test results, including changes in SHBG, serum testosterone, serum glucose, and insulin levels, serum lipid profile, and HOMA-IR values, were observed between the experimental and control groups at 8 weeks (Table 6).

### Safety Assessment

No statistically significant differences in the BMI were observed between the experimental and control groups during either period or group comparisons. Additionally, no changes were observed in the CBC, BUN/Cr, PSA levels, and factors in urine analysis between the 2 groups (data not shown).

## Discussion

Aging male syndrome (also known as andropause) is a clinical and biochemical syndrome characterized by the symptoms of aging and serum testosterone deficiency. Typical symptoms include decreased libido, impaired sexual function, depression, and lethargy. However, unlike menopause in women, symptoms do not appear rapidly in men and do not affect all men. Excluding testicular aging, factors that can contribute to a decline in testosterone levels include excessive drinking and smoking, chronic stress, poor nutritional status, obesity, hypertension, and various chronic conditions such as diabetes, hyperlipidemia, and liver disease. Unhealthy dietary habits and lifestyle are also important causes of andropause.^13^

Restoring the decline in testosterone levels is the primary goal of treating andropathic symptoms. However, testosterone is mostly administered via injection, with only a small portion available in transdermal or oral forms. This can be challenging for individuals who fear needles. In addition, long-term treatment is necessary to achieve sustained benefits. When taking testosterone over a prolonged period, it is important to monitor for potential risks, such as prostate cancer and thrombosis, and cost, as it can be a concern. Therefore, andropause treatment should prioritize improving the overall quality of life rather than solely treating the condition. Therefore, safer and more convenient treatment options are needed to ensure continued patient satisfaction and improved quality of life. One treatment option is to take natural products that can delay or prevent the development of andropause or increase the serum levels of testosterone.^6,7^

Here, we performed a double-blind, randomized, placebo-controlled, and parallel-group study. The AMS and ADAM questionnaires are internationally well-adopted for andropause diagnosis.^14,15^ Self-diagnostic methods are also used for andropause; in clinical studies, AMS is used to screen patients with andropathy. Park et al^6^ and Lee et al^16^ enrolled subjects with a total AMS score of 27. Although diagnostic methods for andropause include biochemical tests to measure testosterone levels and screen for hyperlipidemia, it is difficult to diagnose andropathy. This is because of 2 reasons: the accepted lower limit for serum testosterone levels and the diagnostic methods for andropause that are influenced by factors such as lifestyle and stress.^14^ Previous study suggested that testosterone levels > 500 ng/dL are effective in healthy males.^17^ Another study showed that males with >500 ng/dL of testosterone do not require any treatment, but patients with 250-350 ng/dL testosterone need testosterone therapy, and periodically measuring serum testosterone levels is recommended in aged males with 300-500 ng/dL testosterone.^18^ Therefore, we enrolled subjects by screening them with a total score of more than 37 on the AMS, which indicates moderate or severe symptoms, rather than with a testosterone level of <350 ng/dL. This clinical study included participants aged 43-70 years who had a total score ≥ 37 on the AMS questionnaire and testosterone level ≤ 500 ng/dL.

Primary efficacy assessment measured the changes in the AMS, ADAM, IIEF, and IPSS scores from the baseline. In the AMS and ADAM questionnaires, the experimental group had significantly improved symptom scores compared to the control group after 8 weeks; however, no significant improvement was observed in the IIEF questionnaire score. The AMS and ADAM scores revealed that all patients in the experimental group reported satisfaction with the treatment compared to the control group, even within a short period of 2 months. Previous studies suggested the alleviating effects of *Eurycoma longifolia* extract (Physta^®^), a mixed extract of *Trigonella foenum-graecum* seed and *L. cuneata*, and a complex of Korean dandelion and rooibos on andropause-related symptoms by presenting the decreased scores of ADAM and AMS.^6,7,19^ Consistent with the results of previous studies, the current study showed the significantly decreased total AMS score and the mean of positive number of all questions in ADAM in the experimental group compared to those in the control group, indicating that LCEP has the ability to improve the health of aged males with andropause. Contrary to the results for AMS and ADAM, no significant differences were observed in the IIEF scores between the control and experimental groups. However, while the control group did not show any significant changes in IIEF scores, the experimental group showed improved performance from with baseline. These findings suggest that the duration of the study may have been too short to observe changes in the IIEF score. Therefore, long-term clinical trials are needed to investigate the effects of LCEP on these parameters.

Secondary efficacy assessment measured the changes in serum testosterone, SHBG, serum fasting glucose, and serum cholesterol (total, LDL, HDL, and triglycerides) levels from the baseline. Additionally, the safety assessment measured the changes in CBC, BUN/Cr, urinalysis, PSA levels, serum glucose levels, and serum lipid profile from the baseline. Changes in major variables, such as total cholesterol, HDL cholesterol, LDL cholesterol, and fasting blood glucose levels, after LCEP ingestion did not differ between the experimental and control groups. Although LCE alleviated aging symptoms and increased serum testosterone levels in aged rats,^12^ no significant differences were observed in the serum testosterone levels in this clinical study. However, LCEP decreased the total scores of AMS and ADAM compared to those in control groups at 8 weeks. These discrepancies may be due to the androgen receptor and dosage of the extract. LCEP may increase the expression of androgen receptors in the testes of subjects. Androgen receptors are nuclear receptors that regulate physiological phenomena, such as spermatogenesis and Leydig cell proliferation, by binding to testosterone, translating in the nucleus, and regulating the transcription of DNA.^20^ Androgen receptor in the testes are used as biomarkers that positively regulate the symptoms of andropause. Several studies have examined the changes in the expression levels of androgen receptors in animals and checked the sequence of androgen receptors in humans.^21-23^ In addition, some pharmaceutical companies have developed androgen receptor regulators.^24^ Oral administration of LCE to rats at 150 and 300 mg/kg/day increased the RNA expression levels of the androgen receptors,^20^ suggesting that LCEP regulates the androgen receptor expression to improve the andropause symptoms in affected patients. The second possible explanation may be the dosage. The dosage of 1.25 g/day of LCEP used in this study is equivalent to approximately 150 mg/kg/day of LCE in rats, according to the calculation method suggested by Shin et al^20,25^ Previous studies had presented the result that, in terms of increasing testosterone, significant effect was shown on the high dosage of LCE at 250 and 300 mg/kg/day to rats, but the low dosage of LCE at 125 and 150 mg/kg/day was not effective to rats.^12,20^ Thus, further clinical trials are necessary to determine whether LCEP regulates the serum levels of testosterone at dosages >2.5 g/day.

*L. cuneata* has many physiologically active substances, such as quercetin, kaempferol, vitexin, pinitol, β-sitosterol, daidzein, and desmosin.^10,11,26^ Phytochemicals exert positive effects on male menopause. For instance, daidzein increases the levels of dehydroepiandrosterone, a testosterone derivative, in middle-aged male Wistar rats.^27^ Quercetin increases the activities of 3β- and 17β-hydroxysteroid dehydrogenase, which are key enzymes in the synthesis of testosterone.^28^ β-Sitosterol acts as an inhibitor of 5α-reductase, blocking the degradation of testosterone to dihydrotestosterone in the prostate of hamsters.^29^ Li et al^30^ reported that vitexin alleviates sexual dysfunction and fertility impairment in streptozotocin-treated male mice by regulating the hypothalamus-pituitary-gonadal axis. Each compound has its own molecular mechanisms affecting spermatogenesis, testosterone content, and fertility impairment. Thus, the phytochemical complex in LCEP exerts the major positive effects in alleviating the andropause symptoms in aged males.

In conclusion, LCEP improved the symptoms of andropause with minimal side effects in this study, making it a promising option for andropause treatment. However, additional clinical trials of longer duration are needed to validate our findings and evaluate the long-term safety and efficacy of LCEP in a larger population.

## Figures and Tables

**Table 1.** Patient Baseline Characteristics

Characteristics	Total		Experiment Group		Control Group		*P* ^a^
	(n = 103)		(n = 52)		(n = 51)		
	Mean	SD	Mean	SD	Mean	SD	
Age (years)	54.8	6.7	55.2	6.1	54.3	7.4	.58
Height (cm)	170.7	5.2	169.6	4.6	171.8	5.6	.37
Weight (kg)	76.9	10.5	75.4	9.2	78.2	11.6	.58
BMI (kg/m^2^)	26.5	3.1	26.2	3.0	26.8	3.2	.94

BMI, body mass index; SD, standard deviation.

^a^*P*-value estimated using chi-square test or *t*-test.

**Table 2.** Changes of Aging Male's Symptom

Characteristics	Period	Experimental		Control		*P* ^a^	*P* ^b^	*P*-interaction^c^
		Group (n = 52)		Group (n = 51)				
		Mean	SD	Mean	SD			
Total score	Baseline	51.8	10.1	52.1	7.2	.83	<.0001	<.0001
	4 weeks	42.8	11.6	50.8	7.3	<.0001		
	8 weeks	34.8	12.1	54.8	6.6	<.0001		
	*P* ^d^	<.0001		.02				
	*P* ^e^	<.0001		.02				
Psychological score	Baseline	14.8	2.9	14.5	2.5	.68	< .0001	< .0001
	4 weeks	12.0	3.8	14.3	2.6	.0006		
	8 weeks	10.8	3.5	15.2	2.4	< .0001		
	*P* ^d^	.01		.001				
	*P* ^e^	<.0001		.003				
Somatic score	Baseline	20.8	4.7	21.4	4.0	.46	< .0001	< .0001
	4 weeks	17.1	5.5	20.9	3.9	<.0001		
	8 weeks	14.3	5.4	21.6	4.1	<.0001		
	*P* ^d^	.0004		.01				
	*P* ^e^	< .0001		.40				
Sexual score	Baseline	16.2	3.7	16.2	2.6	.93	< .0001	< .0001
	4 weeks	13.8	3.8	15.6	2.7	.01		
	8 weeks	11.7	4.2	16.3	2.5	< .0001		
	*P* ^d^	.0002		.0001				
	*P* ^e^	< .0001		.38				

SD, standard deviation.

^a^Compared between groups: *P*-value by *t*-test.

^b^Compared between groups: *P*-value by repeated measure analysis of variance.

^c^Compared between groups: *P*-interaction (time × treatment) by repeated measure analysis of variance.

^d^Compared between baseline and 4 weeks: paired *t*-test.

^e^Compared between baseline and 8 weeks: paired *t*-test.

**Table 3.** Change of Androgen Deficiency in Aging Males Questionnaire Positive Rate

Characteristics	Period	Experimental		Control		*P* ^a^	*P* ^b^	*P*-interaction^c^
		Group (n = 52)		Group (n = 51)				
		Mean	SD	Mean	SD			
All question: positive number	Baseline	7.0	2.1	7.4	2.4	.46	.01	.0003
	4 weeks	4.8	3.3	6.5	2.8	.005		
	8 weeks	3.8	3.3	7.0	2.6	<.0001		
	*P* ^d^	<.0001		.003				
	*P* ^e^	<.0001		.19				
Question #1 and 7: positive number	Baseline	1.7	0.6	1.7	0.6	.77	.02	.0005
	4 weeks	1.1	0.9	1.4	0.8	.11		
	8 weeks	0.8	0.9	1.6	0.7	<.0001		
	*P* ^d^	<.0001		.0006				
	*P* ^e^	<.0001		.23				

SD, standard deviation.

^a^Compared between groups: *P*-value by *t*-test.

^b^Compared between groups: *P*-value by repeated measure analysis of variance.

^c^Compared between groups: *P*-interaction (time × treatment) by repeated measure analysis of variance.

^d^Compared between baseline and 4 weeks: paired *t*-test.

^e^Compared between baseline and 8 weeks: paired *t*-test.

**Table 4.** Changes of International Index of Erectile Function

Characteristics	Period	Experimental		Control		*P* ^a^	*P* ^b^	*P*-interaction^c^
		Group (n = 52)		Group (n = 51)				
		Mean	SD	Mean	SD			
IIEF	Baseline	35.6	16.4	37.5	13.1	.52	.69	.15
	4 weeks	40.2	10.4	40.5	16.2	.91		
	8 weeks	43.4	17.5	38.9	15.3	.16		
	*P* ^d^	.005		.03				
	*P* ^e^	<.0001		.20				

IIEF: International Index of Erectile Function; SD, standard deviation.

^a^Compared between groups: *P*-value by *t-*test.

^b^Compared between groups: *P*-value by repeated measure analysis of variance.

^c^Compared between groups: *P*-interaction (time × treatment) by repeated measure analysis of variance.

^d^Compared between baseline and 4 weeks: paired *t*-test.

^e^Compared between baseline and 8 weeks: paired *t*-test.

**Table 5.** Changes of International Prostatic Symptom Score (IPSS)

Characteristics	Period	Experimental		Control		*P* ^a^	*P* ^b^	*P*-interaction^c^
		Group (n = 52)		Group (n = 51)				
		Mean	SD	Mean	SD			
IPSS	Baseline	13.2	6.8	12.9	5.8	.79	.84	.63
	4 weeks	10.4	7.1	10.6	5.7	.85		
	8 weeks	9.5	6.8	10.1	5.4	.67		
	*P* ^d^	.002		.001				
	*P* ^e^	<.0001		.0004				
QOL	Baseline	3.0	1.5	3.1	1.3	.71	.93	.71
	4 weeks	2.8	1.4	2.5	1.4	.28		
	8 weeks	2.3	1.3	2.5	1.4	.37		
	*P* ^d^	.17		.0002				
	*P* ^e^	<.0001		.002				

IPSS: International Prostate Symptom Score; SD, standard deviation; QOL: Quality of life.

^a^Compared between groups: *P*-value by *t*-test.

^b^Compared between groups: *P*-value by repeated measure analysis of variance.

^c^Compared between groups: *P*-interaction (time × treatment) by repeated measure analysis of variance.

^d^Compared between baseline and 4 weeks: paired *t*-test.

^e^Compared between baseline and 8 weeks: paired *t*-test.

**Table 6.** Secondary Effect of Experiment Subject

Characteristics	Period	Experimental			Control			*P* ^a^	*P* ^b^	*P*-interaction^c^
		group (n = 52)			group (n = 51)					
		Mean		SD	Mean		SD			
Cholesterol, total (mg/dL)	Baseline	183.7		43.5	194.4		38.2	.19	.01	.77
	4 weeks	180.4		43.9	191.1		37.6	.19		
	8 weeks	184.2		45.4	198.2		38.8	.09		
	*P* ^d^	.21			.19					
	*P* ^e^	.88			.23					
Cholesterol, HDL (mg/dL)	Baseline	46.1		10.0	51.6		14.6	.03	<.0001	.60
	4 weeks	45.4		10.0	52.2		14.7	.01		
	8 weeks	45.3		9.7	52.7		15.5	.00		
	*P* value^d^	.40			.48					
	*P* value^e^	.36			.26					
Triglyceride (mg/dL)	Baseline	189.6		122.2	183.2		167.3	.83	.14	.71
	4 weeks	209.2		130.4	172.5		93.3	.10		
	8 weeks	201.9		111.0	182.5		97.5	.35		
	*P* ^d^	.12			.49					
	*P* ^e^	.47			.98					
Testosterone (ng/mL)	Baseline	3.9		0.8	3.9		0.7	.80	.04	.46
	4 weeks	3.7		1.1	4.1		1.0	.06		
	8 weeks	3.8		1.0	4.1		1.0	.25		
	*P* ^d^	.11			.22					
	*P* ^e^	.61			.28					
SHBG (nmol/L)	Baseline	35.3	10.7		35.2	9.8		.97	.33	.61
	4 weeks	33.3	9.8		35.6	10.8		.27		
	8 weeks	35.2	11.8		36.7	11.6		.54		
	*P* ^d^	.006			.66					
	*P* ^e^	.97			.11					
Fasting blood glucosee	Baseline	103.9	16.7		96.9	16.3		.03	.01	.62
	4 weeks	103.8	18.8		100.0	16.2		.28		
	8 weeks	102.0	17.8		97.3	14.6		.14		
	*P* ^d^	.96			.24					
	*P* ^e^	.19			.81					
Insulin	Baseline	11.4	11.5		11.8	8.3		.84	.45	.87
	4 weeks	15.2	12.5		19.7	34.0		.38		
	8 weeks	12.1	9.4		11.6	14.5		.87		
	*P* ^d^	.11			.07					
	*P* ^e^	.72			.93					
HOMA-IR	Baseline	2.9	2.9		2.9	2.0		.86	.55	.89
	4 weeks	4.0	3.7		5.5	11.3		.37		
	8 weeks	3.1	2.7		2.8	3.5		.64		
	*P* ^d^	.09			.08					
	*P* ^e^	.70			.96					

HDL: high-density lipoprotein; HOMA-IR: homeostatic model assessment of insulin resistance; SD, standard deviation; SHBG, sex hormone-binding globulin.

^a^Compared between groups: *P*-value by *t*-test.

^b^ Compared between groups: *P*-value by repeated measure analysis of variance.

^c^Compared between groups: *P*-interaction (time × treatment) by repeated measure analysis of variance.

^d^Compared between baseline and 4 weeks: paired *t*-test.

^e^Compared between baseline and 8 weeks: paired *t*-test.
